# Significance of amebiasis: 10 reasons why neglecting amebiasis might come back to bite us in the gut

**DOI:** 10.1371/journal.pntd.0007744

**Published:** 2019-11-14

**Authors:** Debbie-Ann T. Shirley, Koji Watanabe, Shannon Moonah

**Affiliations:** 1 Department of Pediatrics, University of Virginia School of Medicine, Charlottesville, Virginia, United States of America; 2 AIDS Clinical Center, National Center for Global Health and Medicine, Shinjuku, Tokyo, Japan; 3 Department of Medicine, University of Virginia School of Medicine Charlottesville, Virginia, United States of America; Hitit University, Faculty of Medicine, TURKEY

## Introduction

**Fig 1:**
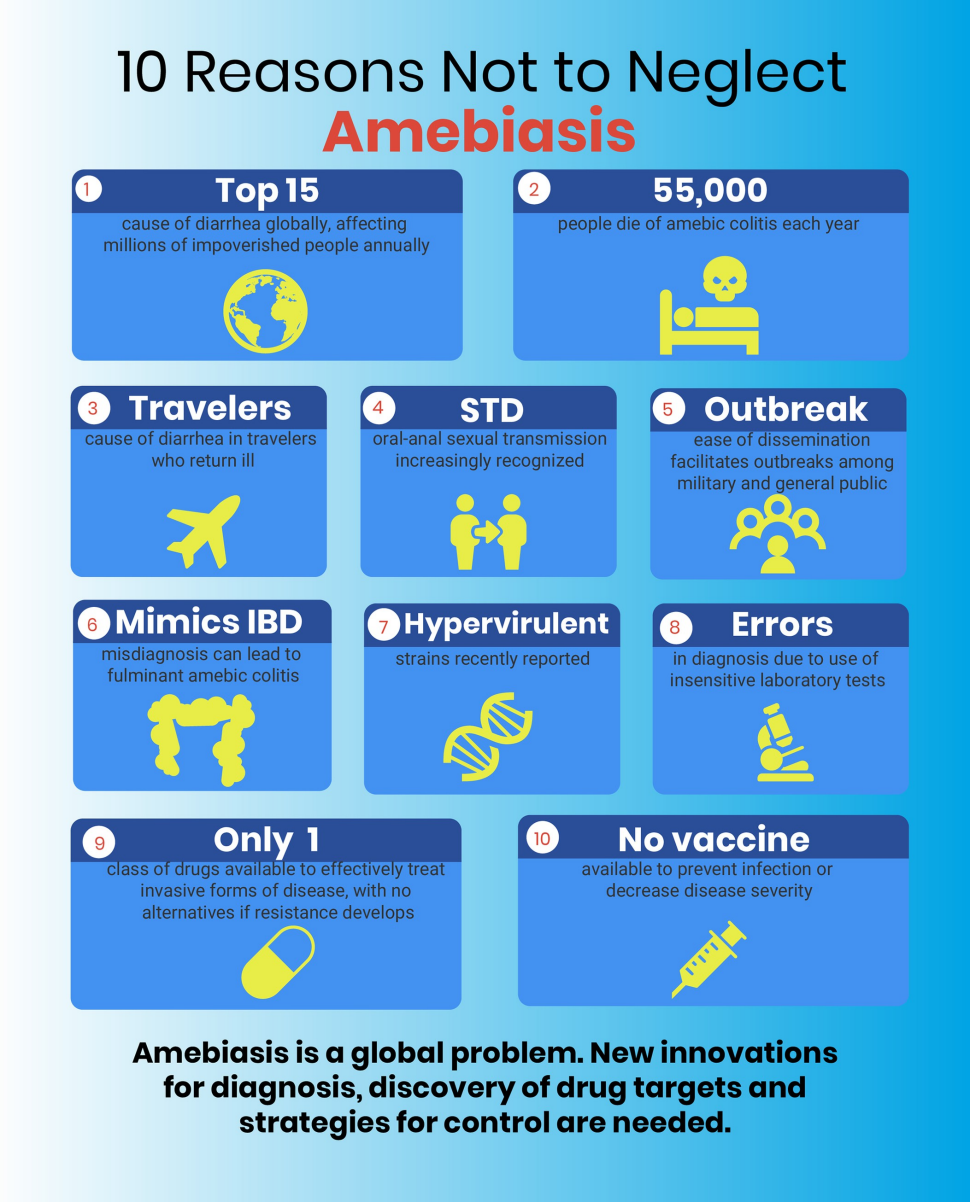
Significance of amebiasis.

Nearly 150 years since the first detailed description of the invasive, tissue-destroying intestinal parasite, *Entamoeba histolytica*, amebiasis remains an infection of consequential global importance. Infection with *E*. *histolytica* can lead to amebic colitis, amebic dysentery, and amebic liver abscess. Even though amebiasis is a leading cause of diarrhea globally, a lack of basic science research to improve our understanding of the complex pathogenesis of this parasite hampers progress. Renewed attention is required to help combat this infection of poverty in order to develop innovative and inexpensive point-of-care diagnostic and surveillance tools, novel treatment options, and effective preventive strategies to help end amebiasis transmission. This viewpoint summarizes 10 reasons why amebiasis is a global health problem in need of further attention (**Fig 1**).

## Reason #1: Amebiasis remains an infection of top global importance, particularly in poverty-stricken settings

Amebiasis is a leading cause of severe diarrhea worldwide [1, 2], though estimates of actual disease burden may be prone to reference test bias, so some heed is warranted. In a large, multinational, prospective case-based study of children with moderate-to-severe diarrhea using molecular methods to identify etiology, however, amebiasis ranked among the top 15 causes of diarrhea in the first two years of life in children living in developing countries, where diarrhea remains the fifth leading cause of death in children under the age of five years [1, 3–5]. While amebiasis occurs worldwide, it is largely an infection of impoverished communities, particularly when sanitation is poor. Amebiasis remains endemic in several developing areas of Central and South America, Asia, and Africa [2]. Advances in molecular technology have improved our understanding of this infection by leading to the recognition and separation of *E*. *histolytica* from other morphologically identical but less-pathogenic or nonpathogenic species of *Entamoeba*, including *E*. *dispar*, *E*. *moshkovskii*, and *E*. *bangladeshi* [6]. Despite the ability to distinguish *E*. *histolytica* by molecular methods, prevalence data on amebiasis remain scarce and imprecise because of inadequate utilization and access to surveillance and diagnostic tools with superior sensitivity and specificity. Some reports that attempt to describe the incidence and prevalence of amebiasis may be inaccurate, especially if the poorly sensitive method of microscopy is used. Experts estimate that millions of people continue to be infected with *E*. *histolytica* each year, and there are several recent reports that help to illustrate the current disease burden of amebiasis. In Mexico, for example, over 8.8 million cases of amebiasis were reported to their National Epidemiological Surveillance System between 2000–2010 [7].The seroprevalence of amebiasis in some rural areas of Mexico reaches as high as 42% [8, 9], although it should be noted that detectable antibodies to *E*. *histolytica* may persist for years, so seroprevalence may overestimate the true disease burden. In Asia, leishmaniasis and amebiasis represent the neglected tropical protozoal infections of highest burden, particularly in the Indian subcontinent [10]. *E*. *histolytica* was detected by molecular methods in nearly 15% of fecal samples submitted for analysis in northeast states of India for instance [11], although again the true disease burden for much of Asia remains unknown. Amebiasis prevalence data from Africa are particularly limited, but it appears widespread. Up to one-third of the population in Vhembe, South Africa had reactive serology, for example [12], while 38% of patients presenting for medical care with acute diarrhea in Egypt were diagnosed with amebiasis by stool antigen study [13]. In the large Global Enteric Multicenter Study (GEMS) of children under the age of five years with moderate-to-severe diarrhea living in seven countries of sub-Saharan Africa and South Asia, *E*. *histolytica* was among the top seven pathogens causing dysentery [4].

## Reason #2: Re-emergence of amebiasis in developed countries

Traditionally, the incidence of amebiasis has been low in industrialized and developed countries, but recent trends have shown reemergence associated with travel to endemic areas, immigration, and sexual transmission [14]. For example, in a large case series from Paris, France; all of the 90 patients with amebic liver abscess identified between the years 2002–2006 were imported, with about half occurring in European-born travelers to tropical areas and the other half in foreign-born immigrants [15]. Amebiasis was the third most frequently isolated pathogen among returning travelers presenting to one of 42 GeoSentinel Surveillance Network sites globally and seeking medical attention for gastrointestinal infection [14]. Travelers to South Asia, the Middle East, and South America appear to be at highest risk, particularly those engaging in missionary and other types of volunteering work [14]. Other reports indicate that a smaller proportion of travelers are affected by amebiasis, and these differences may reflect variations in the epidemiologic risk of groups studied, methods used for detection, duration of travel, and duration of symptoms prior to performing diagnostic testing [16–18]. Here in the United States, the prevalence of amebiasis is about 4%, and, surprisingly, at least five people die in the US from this infection each year [19]. In the state of California, an average of 329 cases of amebiasis are reported annually [20]. In the state of Texas, there are nearly 200 cases of amebiasis reported each year [21]. These two instances in the US underscore an underappreciated problem affecting the poor living among the wealthiest nations, where amebiasis represents one of the most common neglected tropical infections affecting people living in developed countries [22]. Awareness of amebiasis in these settings is important, as lack of familiarity with this infection has led to failure to recognize amebic colitis with resultant fulminant and even fatal outcomes [23].

## Reason #3: Amebiasis is also a sexually transmitted disease

Transmission of amebiasis is through ingestion of the infective cyst, often through fecally contaminated food and water, but direct person-to-person contact, including oral–anal sexual practices, has been recognized increasingly as an alternative means of transmission [24]. Homosexual men, bisexual men, and other groups of men who have sex with men (MSM) in particular are at higher risk of acquisition of amebiasis [25], as highlighted by several countries in Asia, Europe, North America, and Australia [24–29]. In Taiwan for example, HIV-infected MSM had 15 times the odds of having infection with *E*. *histolytica* than other risk groups [26]. In the Beijing and Tianjin provinces of China, amebiasis seroprevalence reached as high as 41% among MSM [25]. A report from Canada recently showed that transmission is also associated with heterosexual and female homosexual activity as well [30].

## Reason #4: Amebiasis can be easily transmitted in outbreak settings

Outbreaks of amebiasis continue to be reported among military and general populations [31, 32]. In a recent outbreak of amebiasis in the Meru county of central Kenya, at least 38 people were hospitalized with four fatalities [33]. Several properties of the *E*. *histolytica* cyst, such as low infectious dose and relative resistance to chlorine, facilitate the ease with which it can be disseminated through contamination of food and water supplies, even in low incidence areas. Thus, *E*. *histolytica* is classified as a category B priority biodefense pathogen by the National Institute of Allergy and Infectious Diseases.

## Reason #5: Severe amebic disease is associated with high fatality

Following ingestion, infective cysts transform to invasive trophozoites, which leads to the development of mucosal inflammation and colonic ulcers [34]. Most of those infected will have asymptomatic amebiasis. For poorly understood reasons, about 10%–20% will develop symptomatic disease, characterized by diarrhea and dysentery. Invasion and dissemination to extraintestinal sites such as the liver can follow. Fulminant disease is rare, occurring in 1%–2% of infections, but carries a high fatality, exceeding 50% in those with severe colitis [23]. *E*. *histolytica* was one of the top 10 causative agents of moderate-to-severe diarrhea in children under the age of five years at two of the GEMS study sites in Bangladesh and Mali. Diarrhea with *E*. *histolytica* was associated with a relatively greater risk of death across all GEMS sites and was the enteric pathogen with the highest hazard ratio for death in the second year of life [5]. It is estimated that amebic colitis kills more than 55,000 people each year [35]. While the incidence of amebic liver abscess is much lower than intestinal amebiasis, the associated morbidity and fatality can also be quite high [15, 36].

## Reason #6: Devastating consequences may arise if amebic colitis is misdiagnosed as inflammatory bowel disease

Patients with amebic colitis may present acutely or chronically with abdominal pain, diarrhea, bloody stools, and weight loss [2]. Many of these symptoms overlap with those of inflammatory bowel disease, and the two conditions may be indistinguishable even by stool inflammatory markers, imaging, endoscopic findings, and lesion distribution [23]. Patients with both symptomatic and asymptomatic forms of amebiasis treated with corticosteroid therapy are at high risk of developing severe and even fatal amebic colitis [23]. This is complicated by the fact that corticosteroids are a mainstay in the management of inflammatory bowel disease, so it is important to consider the diagnosis of amebiasis in patients prior to the administration of corticosteroid treatment. In Turkey, for example, the prevalence of *E*. *histolytica* detected by stool antigen assay was 32% in patients presenting with ulcerative colitis, underscoring the importance of considering this diagnosis particularly in those who reside in endemic areas [37]. Caution is needed here in the face of the increasing global incidence and prevalence of inflammatory bowel disease [38].

## Reason #7: New emergence of hypervirulent strains of *E*. *histolytica*

Of grave concern has been the report of the highly virulent nature of the *E*. *histolytica* strains that were being transmitted in the recent outbreak of amebiasis in Canada [30]. There were substantial differences noted from other *E*. *histolytica* isolates [30]. It is possible that the noted genetic alterations could have either allowed the parasite to more readily evade host immune responses or be associated with higher virulence, resulting in more severe clinical presentations. Ongoing vigilance is needed to identify other hypervirulent strains.

## Reason #8: Inaccurate tests continue to be used for diagnosis

Despite advances in methodologies to diagnose infection with *E*. *histolytica*, lack of access prohibits the use of these more accurate tests. Recommended diagnostic modalities available to assist with diagnosing amebiasis, include molecular assays, stool antigen detection assays, and serology. Molecular assays (considered the gold standard for diagnosis) have high sensitivity and specificity approaching 100%, can be combined with multiplex panels to detect multiple enteric pathogens simultaneously, and can also be used for both stool and fluid samples [2, 39]. Molecular methods, however, are expensive and require specialized instruments and kits for analysis, as well as trained personnel, making this diagnostic method virtually inaccessible to endemic, resource-limited settings. The stool antigen detection tests also have very good sensitivity and specificity in identifying intestinal amebiasis but are expensive, also require expertise to perform, and remain heavily underutilized in endemic areas [2]. Serology can be a useful adjunctive test, particularly for the diagnosis of amebic liver abscess and other extraintestinal manifestations of the disease, but antibodies remain detectable for years after treatment, making it difficult to distinguish between active and past infection [2, 8]. While stool microscopy is more widely available, visualization of cysts and trophozoites can be easily missed by the examiner and when seen cannot be differentiated from other *Entamoeba* species [2, 40]. Stool microscopy has a sensitivity of <60%, and its use should be avoided when other modalities are available [2]. This highlights the need for accessible, inexpensive, point-of-care tests that can diagnose intestinal and extraintestinal forms of amebiasis with high sensitivity and specificity in endemic areas in order to identify those in need of treatment and decrease spread by asymptomatic carriers.

## Reason #9: Lack of drug development makes us ill-prepared to deal with the development of potential resistance

All patients with amebiasis need treatment to mitigate disease and prevent spread, but only a small number of drugs are available to effectively do this. Treatment of amebiasis is complicated, and there is no single agent available that reliably treats both the invasive and intestinal carriage stages of infection. Hence it is recommended that those suffering with symptomatic disease require treatment with two different drugs, an amebicidal tissue-active agent followed by a luminal cysticidal agent. This combination may result in fewer parasitologic failures than use of a tissue-active agent alone [41]. Those with asymptomatic amebiasis only require treatment with a luminal cysticidal agent. A single class of drugs, the nitroimidazoles, currently serve as the mainstay of effective treatment for symptomatic forms of amebiasis [41, 42]. Toxicity can be associated with this class, including peripheral neuropathy, encephalopathy, and cerebellar ataxia [41, 43]. The nitroimidazoles, namely metronidazole and tinidazole, are also the treatment of choice for other anaerobic protists infections, such as giardiasis and trichomoniasis. Given that resistance to these agents has already been documented among *Trichomonas vaginalis* and *Giardia lamblia* [44–47] and the ease of generating resistance strains in the laboratory [48], it is feared that it is only a matter of time before we start seeing the emergence of drug resistant amebiasis. Laboratory induced metronidazole resistant *E*. *histolytica* has already been described through increased expression of iron-containing superoxide dismutase and peroxiredoxin and decreased expression of ferredoxin 1 and flavin reductase, for example [49]. The thiazolide agent, nitazoxanide, has been used by some to treat the tissue invasive from of amebiasis, based on the results of a small, single-center trial conducted in Cairo, Egypt [50]. The high response rate in the placebo arm raises methodologic concerns, and further studies are needed to clarify efficacy before nitazoxanide can be widely recommended as a treatment option for amebiasis. Drugs with cysticidal activity for the treatment of luminal carriage include paromomycin, iodoquinol, and diloxanide furoate. Diloxanide furoate is of very limited availability and significant safety concerns exist with the use of iodoquinol, which has been associated with the development of optic neuritis and peripheral neuropathy [51]. The revival of drugs historically found to have antiamebic activity, such as anisomycin and prodigiosin, and the repurposing of existing drugs already approved for other therapeutic indications with secondary antiamebic properties, such as mefloquine, are alternative strategies currently under active investigation [52]. Furthest along into clinical drug development among these repurposed drugs is the gold compound, auranofin, which targets thioredoxin reductase in *E*. *histolytica*, rendering it susceptible to oxidative stress. Auranofin was previously used in the management of selected patients with rheumatoid arthritis, but further safety analysis is needed to inform whether established concerns such as diarrhea, rash, and bone marrow toxicity will limit its use as an antiparasitic agent [53]. Lastly, several bioactive natural compounds, such as flavinoids and curcumins, have potential antiamebic effect but are much in need of further characterization and are a long way from potential clinical application [54], as recently reviewed [52]. There has really been little progress made in drug development over the past 60 years [55], despite priorities set by the National Institute of Allergy and Infectious Diseases to develop drugs for the treatment of category B biodefense pathogens [52]. The ability to successfully treat amebiasis in the future is unpredictable, and there is an urgent need for effective therapies.

## Reason #10: There is no effective vaccine for the prevention of amebiasis

Partially protective acquired host immunity has been demonstrated, but the relative importance of mucosal, cellular, and humoral immunity in protection is still undetermined. Gal-lectin based vaccinations appear promising in animal models [56]. Other amebic antigenic virulence factors, such as the serine-rich *E*. *histolytica* protein, hold potential as vaccine candidates but are also a long way from clinical development for use in humans, as recently reviewed [52]. Without an effective vaccine, control relies on targeting prevention of transmission by fecal–oral spread. This is quite a daunting task when considering that 2.3 billion people in this world still do not have basic sanitation facilities such as toilets or latrines, and 1.8 billion use a source of drinking water contaminated with feces [57].

## Opportunities for progress

Modest progress in the field has improved our understanding of host cell death, mucosal inflammation, and parasite invasion with amebic infection, but there is still much that is unknown [34, 58–61]. Further understanding of pathogenesis and host response is needed as a way forward towards enhanced treatment and prevention strategies. Increased resources should be devoted to amebiasis research with priority areas highlighted in **Table 1**. These recommendations overlap with several of those made by the World Health Organization over 20 years ago, overall reflecting how little advancement there has been over the past couple of decades [62]. Greater awareness and advocacy are needed to develop funding and interest to create multitargeted approaches designed to help break the cycle of transmission and prevent severity of disease. Although amebiasis is estimated to be responsible for 2.2 million disability-adjusted life years, it not listed in the Global Funding of Innovation for Neglected Diseases (G-Finder) Report indicating negligible funding [63, 64]. Now is the time to create opportunities to build on the recent interest and success in helping to improve global health outcomes.

**Table 1:** Priority areas for amebiasis research.

1. **Improved use of molecular epidemiology for surveillance to understand the true prevalence of amebiasis in developing countries**
2. **Development of inexpensive, easy to use rapid diagnostic tests appropriate for field use in developing countries**
3. **Improved understanding of molecular biology, pathogenesis, and host immune responses to inform control strategies**
4. **Drug development to find safe, effective alternative treatment options**
5. **Vaccine development to identify safe and effective vaccine candidates**
**6. Development of technologies to improve access to clean water and strategies to improve water infrastructure, as well as adequate sewage disposal**

## Conclusions

As summarized in **Fig 1**, the significance of amebiasis is a global problem, with prevalence that may reach as high as 40% in some areas of the world. Disease can be severe and fatal in some, but there are no accurate disease prevalence estimates to help define the true burden. Transmission is fecal–oral and so cannot easily be prevented when there is poverty, inadequate sanitation, and insufficient hygiene. Globalization, immigration, international travel, and sexual practices place everyone at risk of infection. The hardiness of the cyst further lends to ease of spread, predisposing to risks of epidemics among military, the general population, and even as a potential biological threat. The emergence of hypervirulent strains has been newly reported. Treatment options are limited, and there are no other reliably effective options if resistance develops to the nitroimidazole agents, as has already been documented with other protozoan parasites treated by this class of drugs. We still lack a viable vaccine candidate ready to enter into clinical development. Funding agencies dedicated to support research in neglected tropical diseases control should include *E*. *histolytica* as a priority pathogen in order to encourage the development of new innovative technologies for diagnosis, discovery of new drug targets, and strategies for control, including vaccine development. Now is the time to stop neglecting amebiasis.

## References

[1] Estimates of the global, regional, and national morbidity, mortality, and aetiologies of diarrhoea in 195 countries: a systematic analysis for the Global Burden of Disease Study 2016. Lancet Infect Dis. 2018;18(11):1211–28. Epub 2018/09/24. 10.1016/S1473-3099(18)30362-1 30243583 PMC6202444

[2] Shirley DT, Farr L, Watanabe K, Moonah S. A Review of the Global Burden, New Diagnostics, and Current Therapeutics for Amebiasis. Open Forum Infect Dis. 2018;5(7):ofy161 Epub 2018/07/05. 10.1093/ofid/ofy161 30046644 PMC6055529

[3] Current status and progress: Diarrhoea remains a leading killer of young children, despite the availability of a simple treatment solution: UNICEF; Updated June 2018 [cited 2019 April 22]. Available from: https://data.unicef.org/topic/child-health/diarrhoeal-disease/.

[4] Liu J, Platts-Mills JA, Juma J, Kabir F, Nkeze J, Okoi C, et al Use of quantitative molecular diagnostic methods to identify causes of diarrhoea in children: a reanalysis of the GEMS case-control study. Lancet. 2016;388(10051):1291–301. Epub 2016/09/28. 10.1016/S0140-6736(16)31529-X 27673470 PMC5471845

[5] Kotloff KL, Nataro JP, Blackwelder WC, Nasrin D, Farag TH, Panchalingam S, et al Burden and aetiology of diarrhoeal disease in infants and young children in developing countries (the Global Enteric Multicenter Study, GEMS): a prospective, case-control study. Lancet. 2013;382(9888):209–22. Epub 2013/05/14. 10.1016/S0140-6736(13)60844-2 . 23680352

[6] Royer TL, Gilchrist C, Kabir M, Arju T, Ralston KS, Haque R, et al Entamoeba bangladeshi nov. sp., Bangladesh. Emerg Infect Dis. 2012;18(9):1543–5. Epub 2012/08/31. 10.3201/eid1809.120122 22932710 PMC3437720

[7] Bottazzi M, Dumonteil E, Valenxuela J, Betancourt-Cravioto M, Tapia-Conyer R, et al Bridging the innovation gap for neglected tropical diseases in mexico: Capacity building for the development of a new generation ofantipoverty vaccines. B*ol Med Hosp Infant Mex*; 2011 p. 139–46.

[8] Alvarado-Esquivel C, Hernandez-Tinoco J, Sanchez-Anguiano LF. Seroepidemiology of Entamoeba histolytica Infection in General Population in Rural Durango, Mexico. J Clin Med Res. 2015;7(6):435–9. Epub 2015/04/08. 10.14740/jocmr2131w 25883706 PMC4394916

[9] Alvarado-Esquivel C, Hernández-Tinoco J, Francisco Sánchez-Anguiano L, Ramos-Nevárez A, Margarita Cerrillo-Soto S, Alberto Guido-Arreola C. Serosurvey of Entamoeba Histolytica Exposure among Tepehuanos Population in Durango, Mexico. Int J Biomed Sci. 2015;11(2):61–6. 26199578 PMC4502734

[10] Lobo DA, Velayudhan R, Chatterjee P, Kohli H, Hotez PJ. The neglected tropical diseases of India and South Asia: review of their prevalence, distribution, and control or elimination. PLoS Negl Trop Dis. 5 United States2011 p. e1222 10.1371/journal.pntd.0001222 22039553 PMC3201909

[11] Nath J, Ghosh SK, Singha B, Paul J. Molecular Epidemiology of Amoebiasis: A Cross-Sectional Study among North East Indian Population. PLoS Negl Trop Dis. 2015;9(12):e0004225 Epub 2015/12/04. 10.1371/journal.pntd.0004225 26633890 PMC4669114

[12] Samie A, Barrett LJ, Bessong PO, Ramalivhana JN, Mavhandu LG, Njayou M, et al Seroprevalence of Entamoeba histolytica in the context of HIV and AIDS: the case of Vhembe district, in South Africa's Limpopo province. Ann Trop Med Parasitol. 2010;104(1):55–63. Epub 2010/02/13. 10.1179/136485910X12607012373911 . 20149292

[13] Abd-Alla MD, Ravdin JI. Diagnosis of amoebic colitis by antigen capture ELISA in patients presenting with acute diarrhoea in Cairo, Egypt. Trop Med Int Health. 2002;7(4):365–70. Epub 2002/04/16. 10.1046/j.1365-3156.2002.00862.x . 11952953

[14] Swaminathan A, Torresi J, Schlagenhauf P, Thursky K, Wilder-Smith A, Connor BA, et al A global study of pathogens and host risk factors associated with infectious gastrointestinal disease in returned international travellers. J Infect. 2009;59(1):19–27. Epub 2009/05/31. 10.1016/j.jinf.2009.05.008 . 19552961

[15] Cordel H, Prendki V, Madec Y, Houze S, Paris L, Bouree P, et al Imported amoebic liver abscess in France. PLoS Negl Trop Dis. 2013;7(8):e2333 Epub 2013/08/21. 10.1371/journal.pntd.0002333 23951372 PMC3738465

[16] Van Den Broucke S, Verschueren J, Van Esbroeck M, Bottieau E, Van den Ende J. Clinical and microscopic predictors of Entamoeba histolytica intestinal infection in travelers and migrants diagnosed with Entamoeba histolytica/dispar infection. PLoS Negl Trop Dis. 2018;12(10):e0006892 Epub 2018/10/30. 10.1371/journal.pntd.0006892 30372434 PMC6233926

[17] Connor BA, Rogova M, Whyte O. Use of a multiplex DNA extraction PCR in the identification of pathogens in travelers' diarrhea. J Travel Med. 2018;25(1). Epub 2018/02/03. 10.1093/jtm/tax087 . 29394385

[18] Lertsethtakarn P, Silapong S, Sakpaisal P, Serichantalergs O, Ruamsap N, Lurchachaiwong W, et al Travelers' Diarrhea in Thailand: A Quantitative Analysis Using TaqMan(R) Array Card. Clin Infect Dis. 2018;67(1):120–7. Epub 2018/01/20. 10.1093/cid/ciy040 29351583 PMC6248621

[19] Gunther J, Shafir S, Bristow B, Sorvillo F. Short report: Amebiasis-related mortality among United States residents, 1990–2007. Am J Trop Med Hyg. 2011;85(6):1038–40. 10.4269/ajtmh.2011.11-0288 22144440 PMC3225148

[20] Brown E, Dooley D, Smith K. YEARLY SUMMARIES OF SELECTED GENERAL COMMUNICABLE DISEASES IN CALIFORNIA, 2011–2015. 2018.

[21] Amebiasis. Emerging and acute infectious diseases. Texas Department of State Health Services [cited 2019 April 22]. Available from: https://www.dshs.texas.gov/IDCU/disease/Amebiasis.doc.

[22] Hotez PJ. Ten failings in global neglected tropical diseases control. PLoS Negl Trop Dis. 11 United States2017 p. e0005896 10.1371/journal.pntd.0005896 29267282 PMC5739381

[23] Shirley DA, Moonah S. Fulminant Amebic Colitis after Corticosteroid Therapy: A Systematic Review. PLoS Negl Trop Dis. 2016;10(7):e0004879 Epub 2016/07/28. 10.1371/journal.pntd.0004879 27467600 PMC4965027

[24] Hung CC, Chang SY, Ji DD. Entamoeba histolytica infection in men who have sex with men. Lancet Infect Dis. 2012;12(9):729–36. 10.1016/S1473-3099(12)70147-0 . 22917103

[25] Zhou F, Li M, Li X, Yang Y, Gao C, Jin Q, et al Seroprevalence of Entamoeba histolytica infection among Chinese men who have sex with men. PLoS Negl Trop Dis. 2013;7(5):e2232 Epub 2013/05/23. 10.1371/journal.pntd.0002232 23717699 PMC3662687

[26] Hung CC, Ji DD, Sun HY, Lee YT, Hsu SY, Chang SY, et al Increased risk for Entamoeba histolytica infection and invasive amebiasis in HIV seropositive men who have sex with men in Taiwan. PLoS Negl Trop Dis. 2008;2(2):e175 Epub 2008/02/27. 10.1371/journal.pntd.0000175 18301730 PMC2254204

[27] Watanabe K, Gatanaga H, Escueta-de Cadiz A, Tanuma J, Nozaki T, Oka S. Amebiasis in HIV-1-infected Japanese men: clinical features and response to therapy. PLoS Negl Trop Dis. 2011;5(9):e1318 Epub 2011/09/13. 10.1371/journal.pntd.0001318 21931875 PMC3172195

[28] Escola-Verge L, Arando M, Vall M, Rovira R, Espasa M, Sulleiro E, et al Outbreak of intestinal amoebiasis among men who have sex with men, Barcelona (Spain), October 2016 and January 2017. Euro Surveill. 2017;22(30). Epub 2017/08/12. 10.2807/1560-7917.es.2017.22.30.30581 28797327 PMC5553055

[29] Mook P, Gardiner D, Kanagarajah S, Kerac M, Hughes G, Field N, et al Use of gender distribution in routine surveillance data to detect potential transmission of gastrointestinal infections among men who have sex with men in England. Epidemiol Infect. 2018;146(11):1468–77. Epub 2018/06/21. 10.1017/S0950268818001681 . 29923475 PMC9133680

[30] Salit IE, Khairnar K, Gough K, Pillai DR. A possible cluster of sexually transmitted Entamoeba histolytica: genetic analysis of a highly virulent strain. Clin Infect Dis. 2009;49(3):346–53. 10.1086/600298 . 19580413

[31] Kasper MR, Lescano AG, Lucas C, Gilles D, Biese BJ, Stolovitz G, et al Diarrhea outbreak during U.S. military training in El Salvador. PLoS ONE. 2012;7(7):e40404 Epub 2012/07/18. 10.1371/journal.pone.0040404 22815747 PMC3399860

[32] Barwick RS, Uzicanin A, Lareau S, Malakmadze N, Imnadze P, Iosava M, et al Outbreak of amebiasis in Tbilisi, Republic of Georgia, 1998. Am J Trop Med Hyg. 2002;67(6):623–31. 10.4269/ajtmh.2002.67.623 . 12518853

[33] Mutethia G. One dead, 29 hospitalised after amoebiasis outbreak in tigania west. The Star. 2017.

[34] Ghosh S, Padalia J, Moonah S. Tissue Destruction Caused by *Entamoeba histolytica* Parasite: Cell Death, Inflammation, Invasion, and the Gut Microbiome. Curr Clin Micro Rpt. 2019 Epub 21 Jan 2019. 10.1007/s40588-019-0113-6. PMC6449278 31008019

[35] Lozano R, Naghavi M, Foreman K, Lim S, Shibuya K, Aboyans V, et al Global and regional mortality from 235 causes of death for 20 age groups in 1990 and 2010: a systematic analysis for the Global Burden of Disease Study 2010. Lancet. 2012;380(9859):2095–128. 10.1016/S0140-6736(12)61728-0 . 23245604 PMC10790329

[36] Sharma MP, Dasarathy S, Verma N, Saksena S, Shukla DK. Prognostic markers in amebic liver abscess: a prospective study. Am J Gastroenterol. 1996;91(12):2584–8. Epub 1996/12/01. . 8946991

[37] Ozin Y, Kilic MZ, Nadir I, Tayfur O, Ertas A, Ulker A, et al Presence and diagnosis of amebic infestation in Turkish patients with active ulcerative colitis. Eur J Intern Med. 2009;20(5):545–7. Epub 2009/06/27. 10.1016/j.ejim.2009.05.014 . 19712863

[38] Ng SC, Shi HY, Hamidi N, Underwood FE, Tang W, Benchimol EI, et al Worldwide incidence and prevalence of inflammatory bowel disease in the 21st century: a systematic review of population-based studies. Lancet. 2018;390(10114):2769–78. Epub 2017/10/16. 10.1016/S0140-6736(17)32448-0 . 29050646

[39] Ryan U, Paparini A, Oskam C. New Technologies for Detection of Enteric Parasites. Trends Parasitol. 2017;33(7):532–46. Epub 2017/04/08. 10.1016/j.pt.2017.03.005 . 28385423

[40] Ali IK. Intestinal amebae. Clin Lab Med. 2015;35(2):393–422. Epub 2015/05/26. 10.1016/j.cll.2015.02.009 . 26004649

[41] Gonzales MLM, Dans LF, Sio-Aguilar J. Antiamoebic drugs for treating amoebic colitis. Cochrane Database Syst Rev. 2019;1:Cd006085 Epub 2019/01/10. 10.1002/14651858.CD006085.pub3 30624763 PMC6326239

[42] Marie C, Petri WA Jr. Amoebic dysentery. BMJ Clin Evid. 2013;2013. Epub 2013/09/03. 23991750 PMC3758071

[43] Woodruff BK, Wijdicks EF, Marshall WF. Reversible metronidazole-induced lesions of the cerebellar dentate nuclei. N Engl J Med. 2002;346(1):68–9. 10.1056/NEJM200201033460117 . 11778010

[44] Kirkcaldy RD, Augostini P, Asbel LE, Bernstein KT, Kerani RP, Mettenbrink CJ, et al Trichomonas vaginalis antimicrobial drug resistance in 6 US cities, STD Surveillance Network, 2009–2010. Emerg Infect Dis. 2012;18(6):939–43. 10.3201/eid1806.111590 22608054 PMC3358158

[45] Schwebke JR, Barrientes FJ. Prevalence of Trichomonas vaginalis isolates with resistance to metronidazole and tinidazole. Antimicrob Agents Chemother. 2006;50(12):4209–10. Epub 2006/09/25. 10.1128/AAC.00814-06 17000740 PMC1693974

[46] Paulish-Miller TE, Augostini P, Schuyler JA, Smith WL, Mordechai E, Adelson ME, et al Trichomonas vaginalis metronidazole resistance is associated with single nucleotide polymorphisms in the nitroreductase genes ntr4Tv and ntr6Tv. Antimicrob Agents Chemother. 2014;58(5):2938–43. Epub 2014/02/18. 10.1128/AAC.02370-13 24550324 PMC3993245

[47] Leitsch D. Drug Resistance in the Microaerophilic Parasite. Curr Trop Med Rep. 2015;2(3):128–35. 10.1007/s40475-015-0051-1 26258002 PMC4523694

[48] Ehrenkaufer GM, Suresh S, Solow-Cordero D, Singh U. High-Throughput Screening of. Front Cell Infect Microbiol. 2018;8:276 Epub 2018/08/17. 10.3389/fcimb.2018.00276 30175074 PMC6107840

[49] Wassmann C, Hellberg A, Tannich E, Bruchhaus I. Metronidazole resistance in the protozoan parasite Entamoeba histolytica is associated with increased expression of iron-containing superoxide dismutase and peroxiredoxin and decreased expression of ferredoxin 1 and flavin reductase. J Biol Chem. 1999;274(37):26051–6. Epub 1999/09/03. 10.1074/jbc.274.37.26051 . 10473552

[50] Rossignol JF, Kabil SM, El-Gohary Y, Younis AM. Nitazoxanide in the treatment of amoebiasis. Trans R Soc Trop Med Hyg. 2007;101(10):1025–31. Epub 2007/07/20. 10.1016/j.trstmh.2007.04.001 . 17658567

[51] Drugs for parasitic infections 2nd ed Abramowicz J, editor. New Rochelle, New York:2010.

[52] Nagaraja S, Ankri S. Target identification and intervention strategies against amebiasis. Drug Resist Updat. 2019;44:1–14. Epub 2019/05/22. 10.1016/j.drup.2019.04.003 . 31112766

[53] Capparelli EV, Bricker-Ford R, Rogers MJ, McKerrow JH, Reed SL. Phase I Clinical Trial Results of Auranofin, a Novel Antiparasitic Agent. Antimicrob Agents Chemother. 2017;61(1). Epub 2016/12/27. 10.1128/AAC.01947-16 27821451 PMC5192119

[54] Mori M, Tsuge S, Fukasawa W, Jeelani G, Nakada-Tsukui K, Nonaka K, et al Discovery of Antiamebic Compounds That Inhibit Cysteine Synthase From the Enteric Parasitic Protist Entamoeba histolytica by Screening of Microbial Secondary Metabolites. Front Cell Infect Microbiol. 2018;8:409 Epub 2018/12/21. 10.3389/fcimb.2018.00409 30568921 PMC6290340

[55] Debnath A, Parsonage D, Andrade RM, He C, Cobo ER, Hirata K, et al A high-throughput drug screen for Entamoeba histolytica identifies a new lead and target. Nat Med. 2012;18(6):956–60. 10.1038/nm.2758 22610278 PMC3411919

[56] Quach J, St-Pierre J, Chadee K. The future for vaccine development against Entamoeba histolytica. Hum Vaccin Immunother. 2014;10(6):1514–21. Epub 2014/02/06. 10.4161/hv.27796 24504133 PMC5396225

[57] WHO/UNICEF. Progress on drinking water, sanitation, and hygiene. Update and SDG baselines 2017.

[58] Ngobeni R, Abhyankar MM, Jiang NM, Farr LA, Samie A, Haque R, et al Entamoeba histolytica-Encoded Homolog of Macrophage Migration Inhibitory Factor Contributes to Mucosal Inflammation during Amebic Colitis. J Infect Dis. 2017;215(8):1294–302. 10.1093/infdis/jix076 28186296 PMC5853319

[59] Ralston KS, Solga MD, Mackey-Lawrence NM, Somlata, Bhattacharya A, Petri WA. Trogocytosis by Entamoeba histolytica contributes to cell killing and tissue invasion. Nature. 2014;508(7497):526–30. Epub 2014/04/09. 10.1038/nature13242 24717428 PMC4006097

[60] Moonah SN, Jiang NM, Petri WA. Host immune response to intestinal amebiasis. PLoS Pathog. 2013;9(8):e1003489 Epub 2013/08/22. 10.1371/journal.ppat.1003489 23990778 PMC3749964

[61] Thibeaux R, Avé P, Bernier M, Morcelet M, Frileux P, Guillén N, et al The parasite Entamoeba histolytica exploits the activities of human matrix metalloproteinases to invade colonic tissue. Nat Commun. 2014;5:5142 Epub 2014/10/07. 10.1038/ncomms6142 . 25291063

[62] WHO/PAHO/UNESCO report. A consultation with experts on amoebiasis. Mexico City, Mexico 28–29 January, 1997. Epidemiol Bull. 1997;18(1):13–4. Epub 1997/03/01. . 9197085

[63] Policy Cures Research. G-FINDER public search tool 1997 [cited 2019 January 19]. Available from: https://gfinder.policycuresresearch.org/PublicSearchTool/searchDisease.

[64] Murray CJ, Vos T, Lozano R, Naghavi M, Flaxman AD, Michaud C, et al Disability-adjusted life years (DALYs) for 291 diseases and injuries in 21 regions, 1990–2010: a systematic analysis for the Global Burden of Disease Study 2010. Lancet. 2012;380(9859):2197–223. Epub 2012/12/19. 10.1016/S0140-6736(12)61689-4 . 23245608

